# Demographic Features of Invasive Meningococcal Disease in Taiwan, 1993 to 2020, and Genetic Characteristics of Neisseria meningitidis Isolates, 2003 to 2020

**DOI:** 10.1128/spectrum.00882-22

**Published:** 2022-07-11

**Authors:** Chien-Shun Chiou, Ying-Shu Liao, Bo-Han Chen, Min-Chi Lu, Yu-Ping Hong, You-Wun Wang, Ru-Hsiou Teng

**Affiliations:** a Center for Diagnostics and Vaccine Development, Centers for Disease Control, Taichung, Taiwan; b Department of Microbiology and Immunology, School of Medicine, China Medical University, Taichung, Taiwan; University at Albany, State University of New York

**Keywords:** invasive meningococcal disease, *Neisseria meningitidis*, whole-genome sequencing (WGS), multilocus sequence typing (MLST), core genome multilocus sequence typing (cgMLST), molecular epidemiology

## Abstract

We present the demographic features of invasive meningococcal disease (IMD) in Taiwan between 1993 and 2020 and the genetic characteristics of Neisseria meningitidis isolates recovered from 2003 to 2020. IMD was rare in Taiwan between 1993 and 2020, with an annual incidence ranging from 0.009 to 0.204 per 100,000 people. The case fatality rate (CFR) declined from 18.1% for patients in 1993 to 2002 to 9.8% in 2003 to 2020. Infants less than 12 months were most susceptible to the disease. N. meningitidis serogroup B (NmB) was most predominant, responsible for 81.2% (134/165) of the IMD cases in 2003 to 2020. The majority of the isolates recovered from 2003 to 2020 belonged to 4 worldwide-spread hyperinvasive clonal complexes (cc), cc4821 (30.3%), cc32 (19.4%), cc41/44 (12.7%), cc23 (7.3%), and also a newly assigned clonal complex, cc3439 (10.3%). Core genome multilocus sequence typing (cgMLST) profile comparisons revealed that the cc4821 isolates with a T-to-I substitution at position 91 in *gyrA* were closely related to those originating from China. Of the 165 isolates, 20.0% and 53.3% were predicted to be covered by the Bexsero and Trumenba vaccines, respectively, whereas, 77.0% and 46.7% remained indeterminate. In conclusion, N. meningitidis isolates recovered in Taiwan between 2003 and 2020 were mostly highly diverse. Most IMD cases appeared sporadically and were caused by localized strains, although some patients were infected by recently introduced strains. cgMLST is a powerful tool for the rapid comparison of genetic relatedness among a large number of isolates. cgMLST profiling, based on 1,241 core genes, and strain tracking can be performed on the website of cgMLST@Taiwan (http://rdvd.cdc.gov.tw/cgMLST/).

**IMPORTANCE** N. meningitidis can cause life-threatening invasive meningococcal disease (IMD), including meningitis and sepsis, resulting in a high CFR and long-term sequelae in survivors. Here, we report the demographic features of IMD in Taiwan over a 28-year period (1993 to 2020) and the genetic characteristics of N. meningitidis isolates recovered from patients with IMD over an 18-year period (2003 to 2020). We conducted a whole-genome sequence analysis to characterize the genetic features of the isolates and developed a cgMLST scheme for epidemiological investigation and strain tracking. The findings can be beneficial in understanding the epidemiology of IMD in Taiwan, the genetic characteristics of the bacterial strains, and the distribution of vaccine antigens for vaccine development and implementation.

## INTRODUCTION

Neisseria meningitidis is an exclusively human pathogen that colonizes the human nasopharynx, acting as a commensal, but occasionally it can cause life-threatening invasive meningococcal disease (IMD), including meningitis and sepsis, resulting in a high case fatality rate (CFR) and long-term sequelae in survivors (1–3). The incidence of IMD varies markedly over time by country and region, and the majority of countries in the high-incidence group are located in the African meningitis belt (4). IMD occurs most frequently in infants and children aged less than 5 years, and the incidence increases again in adolescents and young adults, even though such an incidence pattern has not been observed in every country (5). Although infants have the highest incidence of IMD, a high CFR is usually observed in adolescents and young adults, and the highest CFR is in the elderly (1, 6). Meningococcal carriage prevalence varies markedly by geographic region and age group, with a peak in adolescents and early adulthood (7–9); the increasing incidence in adolescents may correlate with the nasopharynx colonization rate (9).

N. meningitidis has been classified into at least 12 serogroups, based on the immunologic reactivity of the polysaccharide capsule (10), but only serogroup A (NmA), NmB, NmC, NmW, NmX, and NmY have more often caused life-threatening infections (11). The proportion of meningococcal serogroups causing IMD varies remarkably between geographic regions, countries, and age groups and changes over time (11–13). During 2009 to 2016, NmB was predominant worldwide except in the African region; NmA was primarily circulating in most African countries, Russia, India, and China; NmC was found more frequently in countries of the American region, European region, and the Western Pacific region; NmW was predominant in most African countries and several American and European countries; NmY was detected in higher frequencies in American and European regions and was predominant in Japan (12). As nearly a dozen effective vaccines have been licensed for the prevention of meningococcal disease, surveillance data on serogroup prevalence at the national level are critical for implementing vaccination policies (14).

Many molecular typing methods have been applied to characterize bacterial isolates for the epidemiology study of N. meningitidis infections (15). Multilocus sequencing typing (MLST), which is determined by sequence variations in 7 chosen housekeeping genes, is applicable in determining the clonality, population structure, and global transmission of N. meningitidis clones (16). However, the resolution of MLST is not sufficient in discriminating among closely related strains for a short-term epidemiological study. Multilocus variable-number tandem repeat analysis, which is based on the combination of numbers of repeats in several variable-number tandem repeat loci, provides excellent discriminatory power for highly related strains (17), but this method is difficult to standardize for strain comparisons among laboratories (18). With the advance of next-generation sequencing techniques, whole-genome sequencing (WGS) has become an affordable and powerful tool for characterizing bacterial isolates. WGS has been widely applied in characterizing N. meningitidis isolates in efforts to understand the nature of the pathogen, including genetic diversity, evolution, population structure, and virulence traits (19, 20). WGS data for N. meningitidis can also serve in identifying capsule types (serogroups) (21) and vaccine antigens for vaccine development and implementation (22–25), genetic traits for antimicrobial resistance (26, 27), and the 7-gene MLST profiles and core genome MLST (cgMLST) profiles for epidemiological investigation of global transmission of bacterial clones and disease outbreaks (28). Currently, a cgMLST scheme, comprising 1,605 loci, has been developed for fine typing of N. meningitidis strains (29).

IMD was rare in Taiwan. Between 1993 and 2002, only 4 to 46 IMD cases were confirmed annually in the country, which had a population of around 22 million during the time. In a previous study, Chiou et al. characterized 100 isolates recovered between 1996 and 2002 using pulsed-field gel electrophoresis (PFGE) and MLST (30). The results indicated that NmA, NmC and NmW, NmY, and some NmB isolates belonged to several worldwide-spread hyperinvasive clonal complexes (cc), including cc5, cc11, cc23, and cc41/44. However, 55.8% (29/52) of the NmB isolates belonged to unassigned clonal complexes (ccUA); they could have only existed in Taiwan or some other poorly represented countries. In the present study, we collected demographic data of IMD cases from 1993 to 2020 and characterized N. meningitidis isolates recovered from 2003 to 2020 using WGS, to describe the demographic features of IMD in Taiwan over the past 28 years and the genetic characteristics of isolates from the past 18 years. We also developed a cgMLST scheme for fine typing of isolates for disease outbreak investigation and global strain tracking.

## RESULTS

### Demographic features of IMD.

From 1993 to 2020, a total of 380 IMD cases were reported to the Taiwan Centers for Disease Control (CDC). The annual number of cases ranged from 2 to 46, with a peak in 2002 and an incidence between 0.009 and 0.204 per 100,000 people (see Fig. S1 in the supplemental material). We obtained the demographic data for 371 of the 380 cases from the database of the National Infectious Diseases Surveillance System of the Taiwan CDC. Among the 371 patients, 51 (13.7%) died. The CFR decreased in the most recent 18 years, with CFRs of 18.1% for the cases reported in 1993 to 2002 and 9.8% for 2003 to 2020. Females accounted for 45.6% (169/371) of the IMD cases. Although females had a lower incidence than males (0.054 versus 0.063 per 100,000 people), they had a higher CFR than males (16.0% versus 11.9%). The CFR was particularly high in 2001 (28.2%, 11/39) and 2002 (30.4%, 14/46), and the CFR in that period was much higher in females (39.5%, 17/43) than in males (19.0%, 8/42). Infants aged less than 12 months were most susceptible, accounting for 24.3% (90/371) of the IMD cases; the incidence declined in the age groups of 1 to 19 years but slightly increased in young adults (see Fig. S2). The CFR was higher in the age groups of 20 to 29 years and ≥50 years (see Fig. S3). The disease occurred primarily in the winter and spring seasons and was more prevalent from January to April (see Fig. S4). The nationalities of the patients included Taiwan (366 cases), Thailand (2 cases), Singapore (1 case), Indonesia (1 case), and Australia (1 case). Four patients likely contracted the disease in China, Japan, Indonesia, and/or Singapore.

### Serogroups.

**FIG 1:**
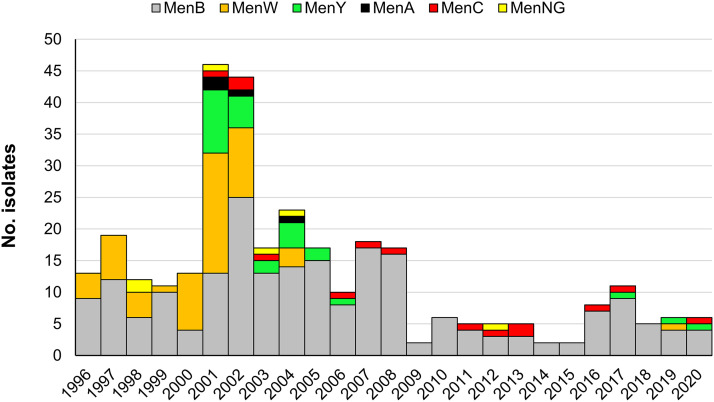
Distribution of serogroups for N. meningitidis isolates in Taiwan, 1996 to 2020 (*n* = 323).

We combined the serogroup data for 158 isolates recovered in 1996 to 2002 and 165 isolates recovered in 2003 to 2020 to demonstrate the distribution of serogroups in the longer term. The isolates in 1993 to 1995 were not collected by Taiwan CDC. The serogroups for the 158 isolates were previously determined using antisera (30), while the serogroups for the 165 isolates were predicted with the WGS data using the tool Meningotype (31). NmA, NmB, NmC, NmW, NmY, and nongroupable (NmNG) accounted for 1.2%, 65.9%, 4.3%, 18.3%, 8.4%, and 1.9% of the 323 isolates, respectively. However, the distribution of serogroups changed over time. NmB, NmC, NmW, and NmY accounted for 50.0%, 1.9%, 34.8%, and 9.5% of the 158 isolates recovered in 1996 to 2002 and 81.2%, 6.7%, 2.4%, and 7.3% of the 165 isolates recovered in 2003 to 2020. NmB was predominant over the 25 years (Fig. 1) and responsible for a higher proportion of infections in 2003 to 2020 than in 1996 to 2002. NmW was prevalent in 1996 to 2002 but was identified in only one patient after 2004. NmA, NmC, and NmY were first identified in 2001. NmA was responsible for only 4 cases that appeared in 2001 to 2004 and was not found again after 2004. NmC was not prevalent but continued causing infections after its emergence in Taiwan in 2001. NmY first emerged in 2001 and caused sporadic infections in 15 persons who were widely distributed across the country in 2001 to 2002 and was responsible for 16.7% (15/90) of the cases during those 2 years, but it was rarely identified after 2006. PFGE and multilocus variable-number tandem repeat analysis indicated that the NmY isolates recovered in 2001 to 2002 were very closely related (17, 30).

### Genetic determinants for ciprofloxacin resistance.

Of the 165 isolates, 15 had a T-to-I mutation at position 91 (T91I) and 1 had a T91A mutation in *gyrA*; none were identified with ciprofloxacin resistance-associated mutations in *parC*. Of the 15 isolates with a T91I mutation, 1 was NmA:cc5, 10 were NmB:cc4821, 2 were NmC:cc4821, 1 was NmB:cc41/44, and 1 was NmB:ccUA. The NmA:cc5 and NmB:cc41/44 isolates appeared in 2004; the first ciprofloxacin-resistant NmC:cc4821 and NmB:cc4821 isolates emerged in 2011 and 2016, respectively.

### Sequence types.

**FIG 2:**
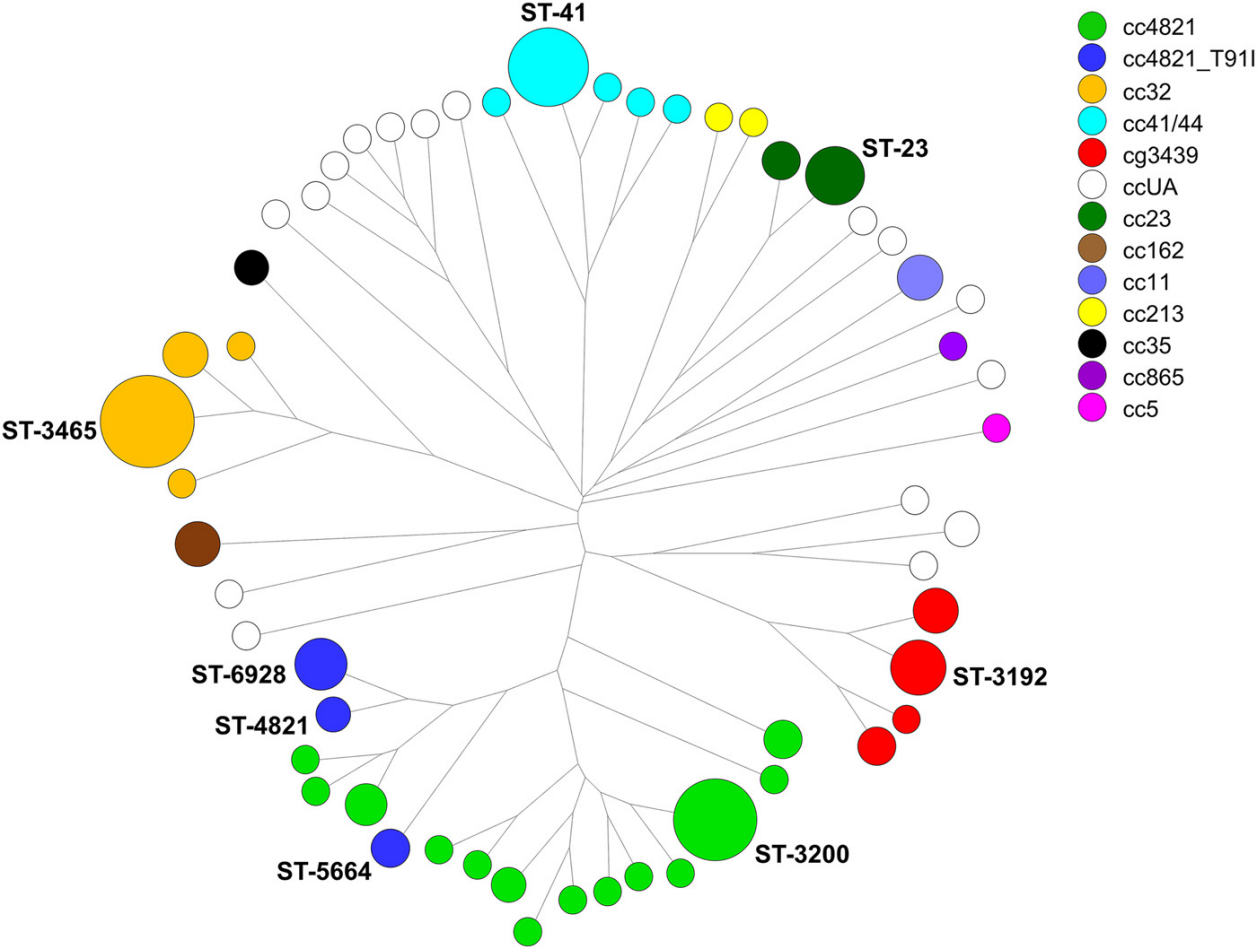
Genetic relationships among 165 N. meningitidis isolates from Taiwan, 2003 to 2020. The genetic tree was constructed with the 7-gene MLST profiles using the neighbor-joining algorithm with the tools provided in BioNumerics version 7.6 (Applied Maths). Each circle represents an ST and is proportional to the number of isolates. Marked texts indicate the major STs for the prevalent clonal complexes and the STs for the isolates with a T91I *gyrA*.

A total of 55 STs were identified in the 165 isolates from 2003 to 2020 (Fig. 2). ST-3465 (25 isolates), ST-3200 (19 isolates), ST-41 (17 isolates), ST-23 (9 isolates), and ST-3192 (8 isolates) were most prevalent among the STs. Of the isolates, 131 (79.4%) fell into 36 STs that belonged to 10 existing clonal complexes, including cc4821 (50 isolates), cc32 (32 isolates), cc41/44 (21 isolates), cc23 (12 isolates), cc162 (5 isolates), cc11 (5 isolates), cc213 (2 isolates), cc35 (2 isolates), cc865 (1 isolate), and cc5 (1 isolate); 17 fell into 4 STs which belonged to a newly designated clonal complex, cc3439, and 17 fell into 16 STs that belonged to ccUA (Fig. 2). Isolates with the same clonal complex were more closely related than those in different clonal complexes. cc4821 was the most prevalent clonal lineage; the 50 cc4821 isolates fell into 17 STs, among which ST-3200 was the dominant type, and all ST-4821, ST-5664, and ST-6928 isolates had a T91I mutation in *gyrA*. Although ST-3200 was a dominant clone of cc4821, the cases caused by this ST dispersed from 2003 to 2020 (see Table S1 in the supplemental material). Similarly, the cases caused by the predominant ST of each clonal lineage, including ST-3465 of cc32, ST-41 of cc41/44, and ST-3192 of cc3439, appeared sporadically over the 18 years (see Table S1). Nevertheless, 2 small outbreaks occurred in a junior high school in 2008 and a military base in 2017, and these were caused by ST-3465:cc32 and ST-6928:cc4821, respectively.

### Meningococcal deduced vaccine antigen reactivity.

Meningococcal deduced vaccine antigen reactivity (MenDeVAR) indexes indicated that 20.0% of isolates were likely covered, 77.0% were indeterminate, and 3% were not covered by the Bexsero vaccine (Table 1). For the Trumenba vaccine, 53.4% of the isolates were likely covered and 46.7% were indeterminate. The data indicated that the vaccine coverage was largely indeterminate for the Taiwanese isolates.

**TABLE 1:** Likely reactivity of meningococcal deduced vaccine antigens of the Bexsero and Trumenba vaccines to N. meningitidis isolates recovered in 2003 to 2020

Serogroup	No. of isolates	Bexsero reactivity				Trumenba reactivity		
		Exact match	Cross-reactive	Insufficient data	None	Exact match	Cross-reactive	Insufficient data
NmA	1	1						1
NmB	134	28	2	103	1	1	74	59
NmC	11			11				11
NmW	4		1		3			4
NmY	12			12			12	
NmNG	3		1	1	1		1	2
Total (%)	165 (100)	29 (17.6)	4 (2.4)	127 (77.0)	5 (3.0)	1 (0.6)	87 (52.7)	77 (46.7)

### cgMLST typing and disease outbreaks.

**FIG 3:**
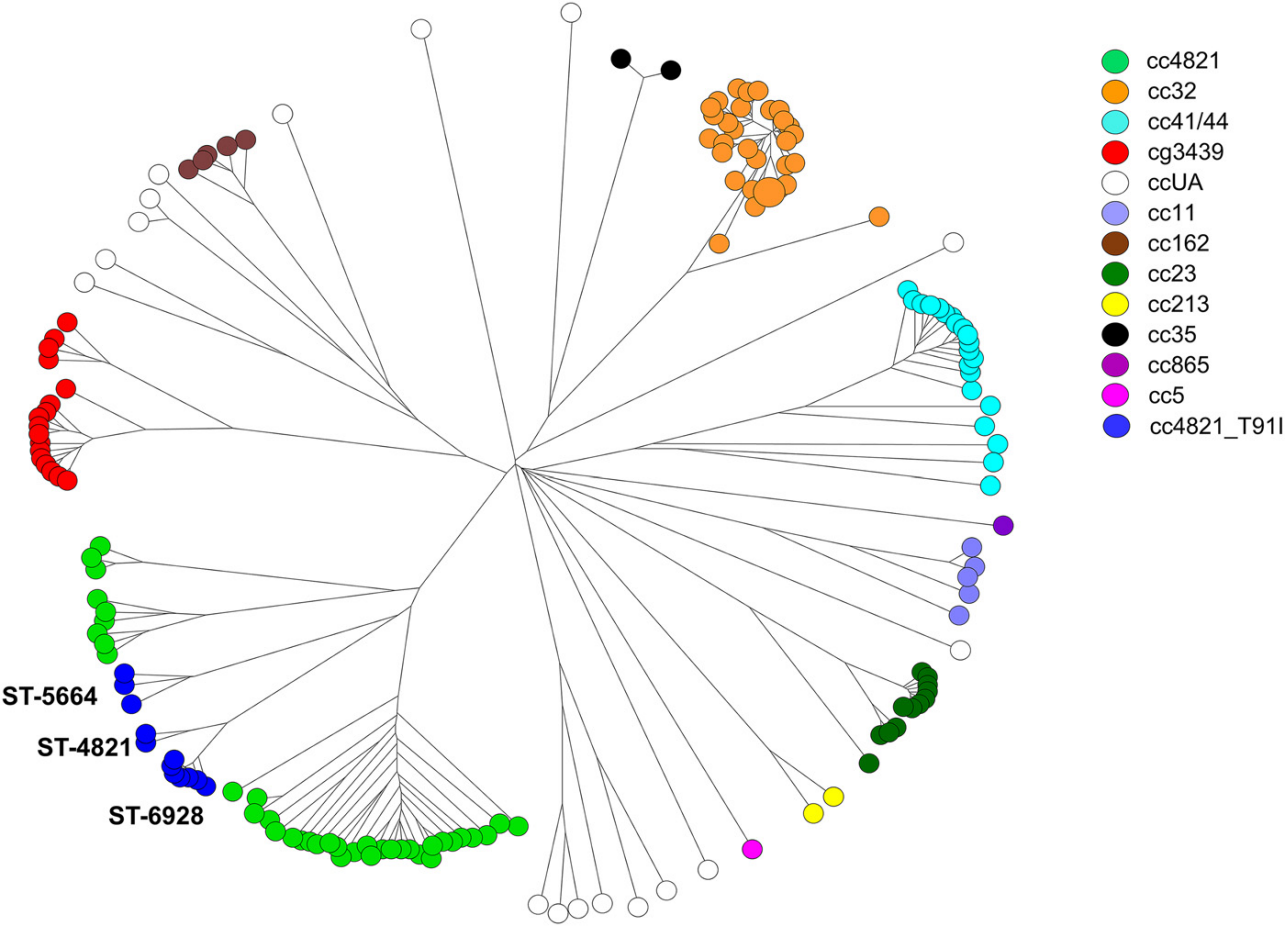
Genetic relationships among 165 N. meningitidis isolates from Taiwan, 2003 to 2020. The genetic tree was constructed using cgMLST profiles and the neighbor-joining algorithm with the tools provided in BioNumerics version 7.6. Each circle represents a cgMLST type and is proportional to the number of isolates.

The cgMLST typing scheme developed in our laboratory comprised 1,241 core genes, which existed in ≥95% of the 319 genomes used for identifying the core genes of N. meningitidis. The cgMLST typing scheme was applied to generate cgMLST profiles for the 165 isolates recovered in 2003 to 2020. A cgMLST tree for the isolates (Fig. 3) revealed a structure of clonal relationships that was similar to that of the MLST tree constructed with the 7-gene-based MLST profiles (Fig. 2). The cgMLST method displayed much higher discriminatory power than the 7-gene-based MLST method; all isolates, except for 3 cc32 isolates, had different cgMLST profiles.

cgMLST analysis confirmed 2 meningococcal disease outbreaks. The first outbreak was reported to have occurred in a junior high school in 2008. Five students were confirmed to have contracted the disease within 4 months (32). Four isolates from the patients were characterized; they differed from each other by only 0 to 2 loci but had a distance of at least 64 loci from other cc32 isolates (see Fig. S5 in the supplemental material). The tight cgMLST cluster included another isolate that was later recovered from an infant residing in the neighboring area. The second outbreak occurred on a military base in northern Taiwan (33). Three soldiers were confirmed to have contracted the disease within weeks. Five isolates from the 3 ill soldiers and 2 adult females residing in northern Taiwan differed by only 1 to 3 alleles (see Fig. S6). These 5 isolates belonged to ST-6928 of cc4821 and had a T91I mutation in *gyrA*. The isolates were previously shown to be ciprofloxacin resistant (33).

### Strain tracking.

We obtained 9,429 genomes from the National Center for Biotechnology Information (NCBI) database and 205 genomes of cc4821 isolates and 141 genomes of cc11 isolates from the PubMLST database for strain tracking of the isolates recovered in Taiwan and comparison with those from other parts of the world. All genomes were converted into cgMLST profiles. The STs, vaccine antigens, and genetic traits for ciprofloxacin resistance for the 9,429 isolates from the NCBI database were identified with the genomic sequences (see Table S2 in the supplemental material). Clustering analysis of cgMLST profiles was conducted to establish the genetic relationships among the isolates of the worldwide-distributed hyperinvasive lineages cc4821, cc32, cc41/44, cc23, and cc11 and the newly assigned cc3439 and ccUA.

**FIG 4:**
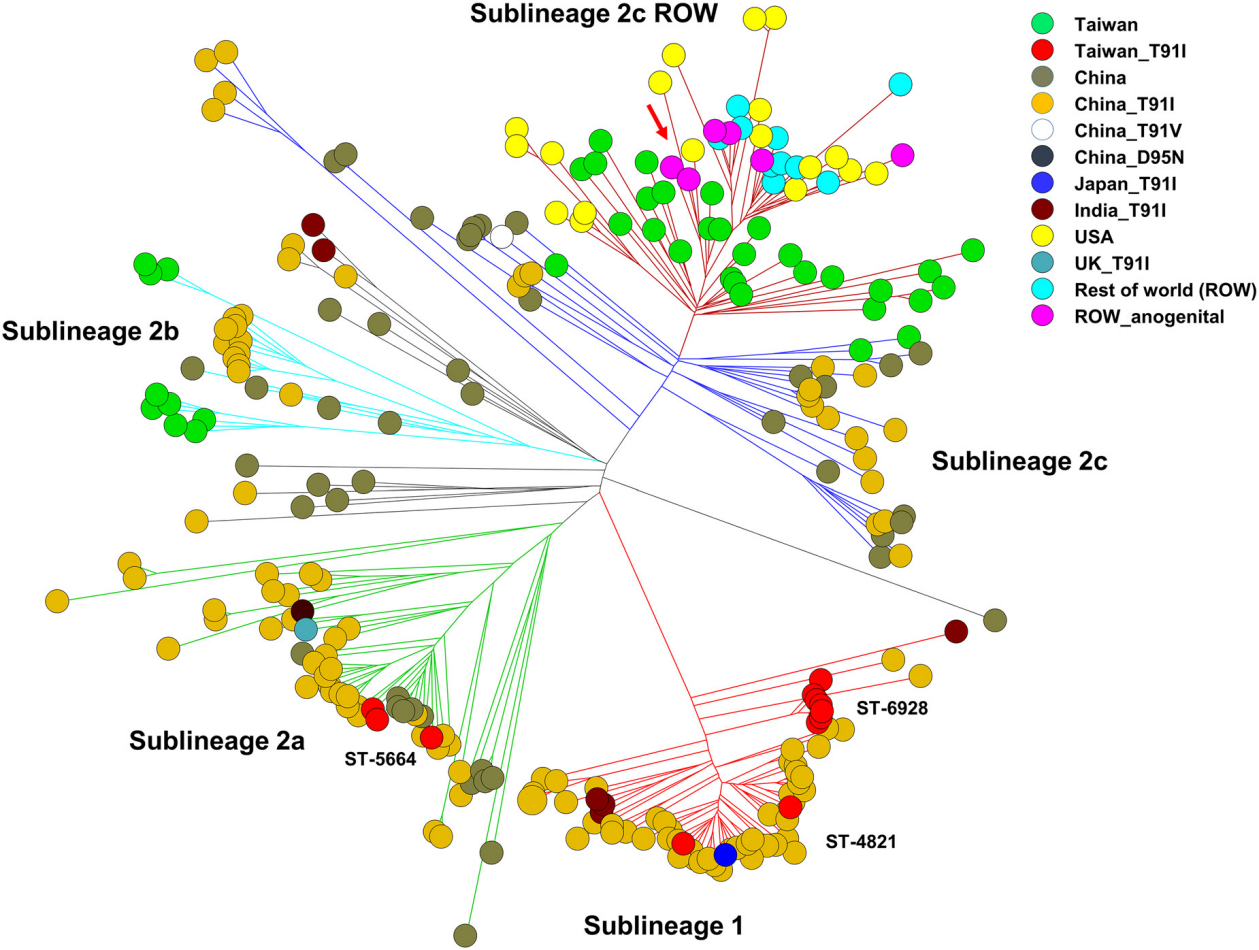
Genetic relationships among cc4821 isolates, constructed with the cgMLST profiles using the neighbor-joining algorithm. The genetic comparison included 50 isolates collected in Taiwan from 2003 to 2020, 188 isolates, including 1 from the USA, that were used in the study of Lucidarme et al. (34), 7 isolates with a T91I substitution in *gyrA* from India and the United Kingdom, and an additional 17 isolates from the United States. The cluster indicated by an arrow comprises 7 isolates, of which 2 were recovered from anogenital sites in men who have sex with men. ROW, rest of the world (Brazil, Greece, Ireland, Italy, Malta, Spain, Sweden, and United Kingdom).

The genetic relatedness among 262 cc4821 isolates (see Table S3) was constructed using cgMLST profiles (Fig. 4). The cgMLST tree indicated that the Taiwanese isolates were distributed among all sublineages, including 1, 2a, 2b, 2c, and 2c ROW, which were defined in the study of Lucidarme et al. (34). The 12 T91I *gyrA*-carrying isolates from Taiwan were distributed in sublineages 1 and 2a, while the 38 isolates with a wild-type *gyrA* were located in sublineages 2b, 2c, and 2c ROW. Notably, 3 isolates in the sublineage 2c ROW were closely related to 2 isolates that were recovered from anogenital sites in men who have sex with men (34). However, the three isolates (R17.0743, R18.2605, and R18.2624) were recovered from female infants of 11 days, 2 months, and 12 months in 2016 and 2018. Of the 12 isolates with the mutation T91I in *gyrA* from Taiwan, 2 were NmC:ST-4821, 3 were NmB:ST-5664, and 7 were NmB:ST-6928. The 2 NmC:ST-4821 isolates were genetically very close to the isolates from China; one of the NmC:ST-4821 isolates was recovered from a patient who contracted the disease in China (Fig. 4). The isolates with a T91I *gyrA* from China were distributed in all sublineages or belonged to diffused isolates. Of the 6 isolates with a T91I *gyrA* from India, 4 were distributed in sublineage 1 and 2 belonged to diffused isolates. The one from the United Kingdom was located in sublineage 2a. All 18 isolates from the USA as well isolates from other 8 countries (ROW) were located in the sublineage 2c ROW (Fig. 4).

The cc32 isolates from NCBI were mostly from the United Kingdom, the United States, the Netherlands, Malta, Ireland, and the Czech Republic (see Fig. S7 in the supplemental material). Of the 32 isolates recovered in Taiwan, 31 were grouped in a cluster distinctly separate from the isolates from other countries, and the remaining one (R20.0333), which emerged in 2019, was located in a group comprising isolates from several countries.

Of the 21 cc41/44 isolates recovered in Taiwan, 16 were grouped tightly in a distinct cluster and the remaining 5 were distributed separately (see Fig. S8). The 5 isolates were also relatively distant from the NCBI isolates, which were mostly from the Netherlands, United Kingdom, and the United States.

Of the 12 NmY:cc23 isolates recovered in Taiwan, 8 that emerged in 2003 to 2005 were ST-23 and were grouped tightly in a distinct cluster that was distantly related to the NmY:cc23 isolates from other countries (see Fig. S9). The NmY:cc23 isolate recovered in 2006 was ST-23 but was distantly related to the 8 ST-23 isolates recovered in 2003 to 2005. The isolate that emerged in 2006 was closely related to a group of isolates mostly from the United States (see Fig. S9). The 3 isolates recovered in 2017, 2019, and 2020 belonged to ST-1655 and were closely related to a large group of isolates mostly from the United Kingdom (see Fig. S9).

**FIG 5:**
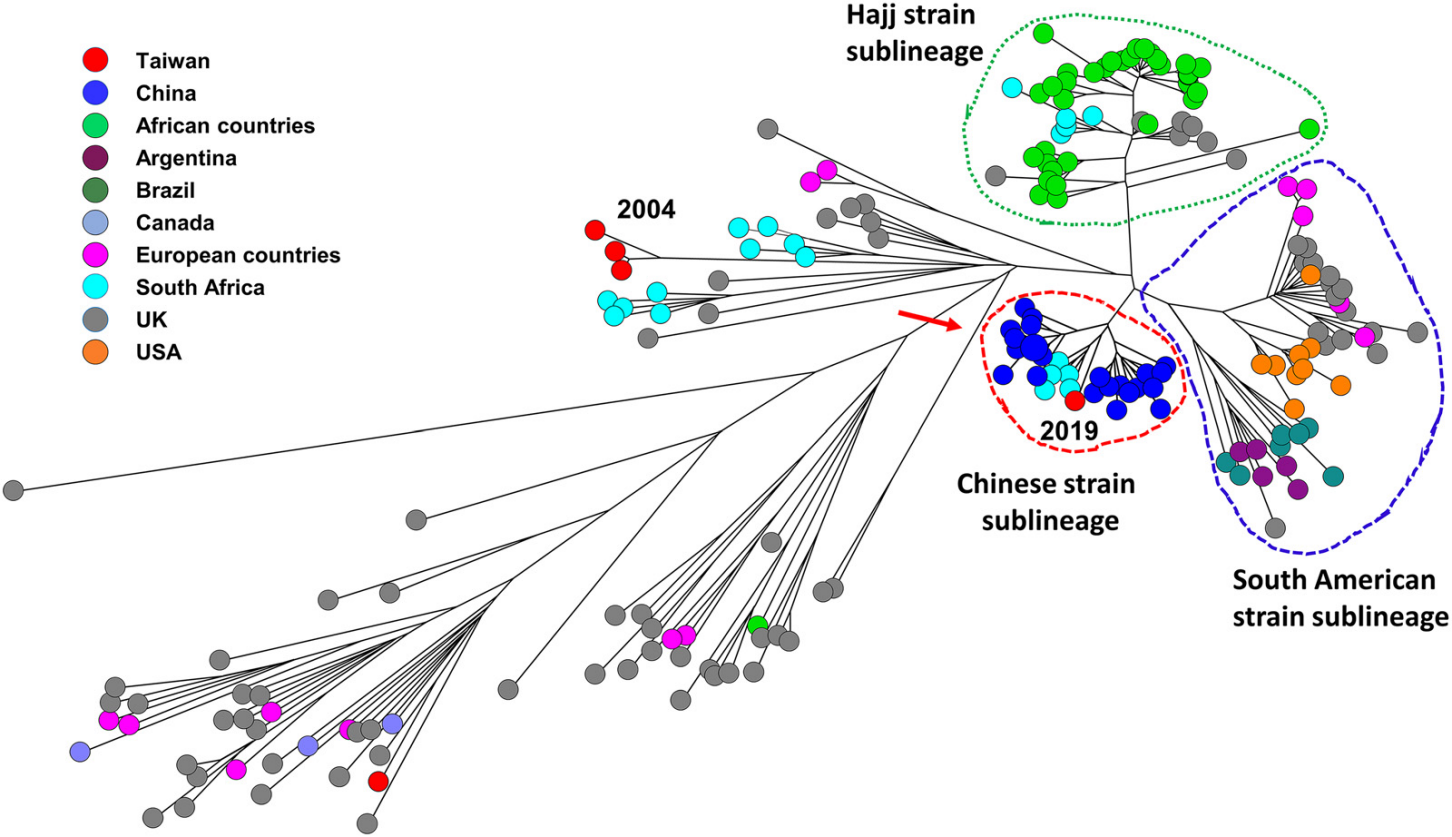
Genetic relationships among cc11 isolates, constructed with the cgMLST profiles using the neighbor-joining algorithm. The genetic comparison included 4 NmW isolates and 1 NmC isolate from Taiwan, 141 NmB, NmC, and NmW isolates that were used in the study of Zhu et al. (35), and 49 isolates belonging to the South American strain sublineage and Hajj strain sublineage from the NCBI database. The African countries included Benin, Burkina Faso, Cameroon, Chad, Ethiopia, Guinea, Mali, and Niger. The European countries included France, Greece, Ireland, and Malta. The years of emergence for the isolates from Taiwan are indicated. The Chinese strain sublineage, Hajj strain sublineage, and the South American strain sublineage are circled by dash lines. The isolates to the right of the red arrow are NmW, while those on the left are NmB and NmC.

Of the 5 cc11 isolates recovered in Taiwan, 4 were NmW and 1 was NmC. The genetic relatedness among 195 cc11 isolates (see Table S4), including 4 NmW and 1 NmC isolates from Taiwan, 141 NmB:cc11, NmC:cc11, and NmW:cc11 isolates used in the study of Zhu et al. (35), and 49 isolates that belonged to the Hajj strain sublineage and the South American strain sublineage from the NCBI database, were established using cgMLST profiles (Fig. 5). The genetic relationships among the cc11 isolates indicated that the 3 NmW:cc11 isolates from Taiwan, recovered in 2004, could be grouped with isolates from South Africa and the United Kingdom, but with a considerable distance (Fig. 5). The rest of the NmW:cc11 isolates, recovered in 2019, were located in the Chinese strain sublineage that was defined in the study of Zhu et al. (35), with a shorter distance to the isolates from South Africa (Fig. 5). Accordingly, of the 4 NmW:cc11 isolates recovered in Taiwan, 3 were genetically distant from and 1 was closely related to, but distinct from, the Hajj strain sublineage and the South American strain sublineage.

The cgMLST profiles for 17 NmB:cc3439 isolates, 15 NmB:ccUA isolates, and 2 NmNG:ccUA isolates from Taiwan were compared with those for the 9,429 isolates from the NCBI database. The comparison indicated that the 34 isolates were all distantly related to those from other parts of the world. However, after cc3439 had been assigned, we found 6 cc3439 strains (1 from the United Kingdom and 5 from South Africa) in the PubMLST database. The five strains from South Africa were grouped in a distinct cluster, whereas the one from the United Kingdom clustered tightly with 13 Taiwanese isolates, which belonged to ST-3192 and ST-5790 (see Fig. S10).

## DISCUSSION

The annual incidence of IMD in Taiwan between 1993 and 2020 ranged from 0.009 to 0.204 per 100,000 people (see Fig. S1 in the supplemental material). Accordingly, Taiwan, as well as many Asian countries, belongs to the country group with low IMD endemicity rates (<2 cases/100,000 population per year) (4, 36). However, the disease has been considered to be re-emerging in the country since the 1990s. The incidence of IMD was high in Taiwan between 1950 and 1968, with an annual incidence that peaked in 1953 (0.94/100,000) (37). The disease nearly disappeared between 1975 and 1987, with only 7 cases reported in that period. Although no serogroup data are available for the bacterial isolates from the IMD cases before the early 1990s, an introduction of NmW strains was likely one of the causes associated with the resurgence of IMD in Taiwan. NmW was responsible for 34.8% of the IMD cases in 1996 to 2002. PFGE analysis indicated that 93.6% (29/31) of the NmW isolates were very closely related, suggesting that the NmW strains were introduced into this country not long before 1996 (30). The dramatic increase in the number of IMD cases in 2001 and 2002 was accompanied by the emergence of NmY, NmA, and NmC strains for the first time in Taiwan in 2001 (Fig. 1). Among these 3 serogroups, NmY strains are most noteworthy, as they were responsible for 16.7% of the IMD cases in 2001 to 2002 and resulted in several deaths.

The CFR declined from 18.1% in 1993 to 2002 to 9.8% in 2003 to 2020. The decline in the CFR should have nothing to do with the vaccine, because no national vaccination program has been implemented in Taiwan since 2002. The decline could be attributed primarily to the high CFR in 2001 and 2002, as 28.2% (11/39) of patients died in 2001 and 30.4% (14/46) died in 2002. In those 2 years, the number of cases abruptly increased, with nearly 4 times the average of the other 26 years (Fig. 1). From 1996 to 2000, all but two cases were caused by NmB and NmW. However, the 2001 to 2002 outbreak was accompanied by the emergence of NmY, NmC, and NmA for the first time in Taiwan. Whether the newly introduced serogroups contributed to the excessive CFR during the period is not known, because we were not able to connect the bacterial isolates and the demographic data for analysis. Another notable feature is that the CFR among females (39.5%) was much higher than for males (19.0%) in those 2 years, but the contributing risk factor was unclear. The finding of females with a higher CFR than males has been observed in New York City, NY, USA (38), but the effect of sex on mortality in IMD was not supported by the results from the surveillance data from 21 European countries (39).

Our data indicate that the CFRs of IMD in Taiwan were higher in the age groups of 20 to 29 years and ≥50 years (see Fig. S3). CFR of IMD varies over age groups. A meta-analysis study indicated that, for laboratory-confirmed IMD cases reported between January 2000 and May 2008, the predicted CFR was high in infants (9.0%), gradually decreased to 7.0% in 7 year olds, subsequently increased to reach a peak of 15.0% in young adults, remained steady in adults aged between 28 and 45 years, and then rose rapidly in older adults (1). Older adults usually have the highest mortality rate of IMD, which could be linked to underlying comorbidities and more atypical presentations hindering appropriate timely diagnosis and management and the dysfunction of the immune response (14, 40). However, little has been discussed on the elevated CFRs in the young adult group. In this study, the increased CFR in the young adult group was contributed by the abrupt increase in the number of deaths between 2001 and 2003; in the past 28 years, 7 of the 9 deaths among patients aged 20 to 24 years occurred in those 3 years. Unfortunately, data are not available to determine if the increased deaths were associated with the newly introduced serogroups (Y, C, and A) in 2001.

The serogroup distributions are considerably different between the N. meningitidis isolates emerging in 1996 to 2002 and those in 2003 to 2020 (Fig. 1). NmB and NmW were most prevalent in 1996 to 2002; they together accounted for 84.8% of the isolates in the period. However, NmW almost disappeared after 2004. The NmW strain that emerged in 2019 was genetically distant from those recovered in 2004 (Fig. 5), suggesting that the circulation of the major NmW clone had ended in 2004. The cause of the disappearance of the NmW clone in Taiwan was unclear, since no national meningococcal vaccination program had been implemented during this period. However, chemoprophylaxis was commonly prescribed for contacts, with immunization being recommended only for those in high-risk groups (36). The rise and fall of a prevalent clone were also observed in a NmY:ST-23 clone (Fig. 1), which first emerged in 2001 in Taiwan and circulated primarily between 2001 and 2005. The NmY:ST-23 isolate from 2006 and the 3 NmY:ST-1655 isolates from 2017 to 2020 belong to two distinct sublineages and are genetically distant from the clone that circulated in 2001 to 2005 (see Fig. S9).

NmB was the most predominant serogroup in Taiwan over the past 25 years. It was found in 50.0% of the isolates recovered in 1996 to 2002, and the proportion increased to 82.4% in 2003 to 2020. The 134 NmB isolates characterized in this study fell into 45 STs, suggesting a great genetic diversity among the NmB isolates. However, the majority (81.3%) of NmB isolates fell into 4 clonal complexes, cc4821, cc32, cc41/44, and cc3439, among which cc4821, cc41/44, and cc3439 were also prevalent in 1996 to 2002. Note that cc32 became prevalent in 2003 to 2020; it was identified in only one isolates recovered in 1996 to 2002 (30).

In a previous study, we indicated that cg3200 should be an endemic clonal lineage unique in Taiwan and should have existed on this island long before 1996 (30). However, strains of this clonal lineage were subsequently identified in China and assigned to cc4821. ST-4821 is the leading epidemic clone of cc4821; it caused numerous NmC outbreaks in Anhui, China, in 2003 to 2005 (41). This clonal lineage soon spread across China (42) and to other countries (43) and has become a predominant clonal group in eastern China (44). In China, cc4821 was first identified in NmC strains, but subsequently, NmB:cc4821 strains emerged through capsular switches (42, 45). In Taiwan, most cc4821 isolates were NmB; the first NmC:cc4821 isolate emerged in 2006 (see Table S5). Clustering analysis of cgMLST profiles of the cc4821 isolates from Taiwan, China, and other countries indicated that the ciprofloxacin-resistant isolates recovered in Taiwan are distributed in sublineages 1 and 2a; the majority of isolates in these two sublineages have a T91I substitution in *gyrA* (Fig. 4). The isolates with a T91I *gyrA* mutation from Taiwan belonged to ST-4821, ST-6928, and ST-5664, which are prevalent in China (42, 45, 46). The isolates with a wild-type *gyrA* from Taiwan are mostly distributed in sublineage 2c ROW and are more closely related to the strains from countries other than China (Fig. 4); most of the isolates in this cluster are ST-3200.

NmW:cc11 had been a prevalent clone in Taiwan from 1996 to 2002 (Fig. 1). PFGE analysis of the NmW isolates emerging in 1996 to 2002 suggested that NmW strains belong to a common clone and could have been introduced into Taiwan not long before 1996 (30). Epidemiological data suggest that the NmW strains circulating in Taiwan from 2001 through 2003 are unrelated to the Hajj strains (47). In this study, by comparing the cgMLST profiles for the 4 NmW:cc11 isolates from Taiwan and other countries, we confirmed that the NmW:cc11 strains that circulated from 1996 to 2004 in Taiwan are distant from the Hajj strain sublineage and the South American strain sublineage, whereas the strain that emerged in 2019 belongs to the Chinese strain sublineage (Fig. 5).

The17 NmB:cc3439 isolates, 15 NmB:ccUA isolates, and 2 NmNG:ccUA isolates do not belong to any existing clonal complex and, to date, are genetically distant from any isolate in the NCBI database; they are likely unique in Taiwan. However, after the ST-3439 clonal complex was assigned, we found 6 cc3439 strains from the United Kingdom and South Africa in the PubMLST database. The one from the United Kingdom is tightly clustered with 13 Taiwanese isolates (see Fig. S10). cc3439 was a major clonal group in Taiwan from 1996 to 2020, but the cc3439 strains are considerably diverse; they should have existed in this country long before 1996 (30).

Interestingly, strain comparisons revealed that the majority of isolates of each worldwide-distributed hyperinvasive lineages (cc32, cc41/44, and cc23) recovered in Taiwan are tightly grouped in a distinct cluster and are distantly related to those from other parts of the world (see Fig. S7, S8, and S9 in the supplemental material). These strains in each clonal complex should have evolved through clonal expansion after introduction into Taiwan.

The MenDeVAR indexes for the Bexsero and Trumenba vaccines are considerably low, with only 20.0% and 53.3% for all isolates and 22.4% and 56.0% for NmB isolates, respectively, whereas the vaccine reactivity in 77% and 46.7% of isolates, respectively, is indeterminate for the two vaccines. In comparison, the predicted coverage for the 4CmenB (Bexsero) vaccine based on the Genetic Meningococcal Antigen Typing System (gMATS) is 23.0% for all isolates and 26.9% for NmB isolates. Thus, the vaccine coverage for the two vaccines is largely indeterminate for the Taiwanese strains.

The N. meningitidis cgMLST scheme developed in our lab comprises 1,241 core genes, but the one developed by Bratcher et al. (29) comprises 1,605 core genes. Of the 1,605 genes, 1,210 correspond to 1,208 core genes identified in our study; 327 correspond to 318 loci in our scheme that are not considered core genes in our study because they are present in <95% of the 319 genomes used in developing our cgMLST scheme, and 68 are not identified as genes by our computational tools. For some loci in our scheme, one locus could correspond to multiple (2 or 3) loci in Bratcher’s scheme. The strains (genome sets) used in developing the cgMLST scheme by Bratcher et al. and our scheme are considerably diverse, but some of the 1,605 loci (339, 21.1%) could not be assigned as core genes if the cutoff value was set at a frequency of ≥95% over a set of 334 genomes from the PubMLST *Neisseria* database (https://pubmlst.org/). The data (figures and tables) discussed in this paragraph and more comparisons regarding the two cgMLST schemes are described further in File S1 in the supplemental material.

Although our cgMLST scheme contains only 1,241 loci, it displays sufficient discriminatory power in distinguishing outbreak isolates from epidemiologically unrelated isolates of the same clonal group (see Fig. S5 and S6). Through the cgMLST analysis, we found 3 additional unidentified patients who were infected with the outbreak strains. This cgMLST approach is also a powerful tool for strain tracking. We converted a large number of NCBI genomes into cgMLST profiles to allow us to compare the genetic relatedness of isolates from Taiwan with those from other parts of the world. We have installed the cgMLST profiling tool and set up a cgMLST profiles database, which contains 9,594 cgMLST profiles of N. meningitidis generated in this study, on a website: BENGA cgMLST@TAIWAN (http://rdvd.cdc.gov.tw/cgMLST/). Each entry in the cgMLST database is accompanied by several genetic traits predicted from the genomic sequence. The web service allows users to generate cgMLST profiles for their isolates by uploading assembled whole-genome sequences and to compare cgMLST profiles with those isolates via the Internet.

In conclusion, IMD was rare and NmB was the most predominant serogroup responsible for the disease in Taiwan. NmB strains were highly diverse and most could be unique or had circulated in Taiwan for a long time. NmW:cc11, NmY:cc23, NmC:cc4821, and some NmB:cc4821, NmB:cc32, and NmB:cc41/44 strains could have been introduced into this country not too long ago. cc4821 is particularly noteworthy, as a large proportion of the cc4821 isolates have developed resistance to ciprofloxacin and likely to some other antimicrobials. The antimicrobial susceptibility in the 165 isolates will be further investigated.

## MATERIALS AND METHODS

### Demographic information and bacterial isolates.

Invasive meningococcal disease is a notifiable disease in Taiwan; hospitals are obligated to report the disease to the Centers for Disease Control, Taiwan. We obtained the statistical numbers of IMD cases from 1993 to 2020 from the National Infectious Disease Statistics System managed by Taiwan CDC (https://nidss.cdc.gov.tw/en/) and the demographic information (gender, age, country of citizenship, county or city of residence, travel history, date of onset, country of infection, symptoms, and age of death) of IMD cases from the Business Object database of Taiwan CDC, under the authorization of Taiwan CDC (IRB110120). We obtained 165 N. meningitidis isolates recovered in 2003 to 2020 from the Biobank Section of the Taiwan CDC. The population statistical data used for the calculation of disease incidence were obtained from the National Statistics, Directorate General of Budget, Accounting, and Statistics (DGBAS) of Executive Yuan, Taiwan (https://eng.stat.gov.tw/mp.asp?mp=5).

### Whole-genome sequencing and analysis.

WGS of bacterial isolates was conducted in the Central Region Laboratory of Taiwan CDC using the Illumina MiSeq sequencing platform. DNA of isolates was extracted using the Qiagen DNeasy blood and tissue kit (Qiagen Co., Germany), library construction was performed using the Illumina DNA Prep Tagmentation system (Illumina Co., USA), and sequencing was run with the MiSeq reagent kit version 3 (2 × 300 cycles). All the procedures were performed by following the manufacturer’s instructions. The WGS reads were subjected to bacterial species identification using the KmerFinder 3.2 provided by the Center for Genomic Epidemiology of the Technical University of Denmark (http://www.genomicepidemiology.org/). Sequence reads were assembled using the SPAdes assembler (48), and the assembled contigs were subjected to the prediction of serogroups, sequence types (STs), PorB, FetA, and Bexsero antigen sequence types (BAST; fHbp, NHBA, NadA, and PorA) using the Meningotype tool (31), which relies on the PubMLST database for the prediction of the antigens and serogroups. To assess the likely reactivity of the meningococcal deduced vaccine antigens of Bexsero and Trumenba vaccines, we used the tool of the MenDeVAR Index (24). The genetic characteristics of 165 isolates obtained from WGS data are listed in Table S5 in the supplemental material. cgMLST profiles were generated using an in-house-developed cgMLST scheme and profiling tool (http://rdvd.cdc.gov.tw/cgMLST/). Mutations were examined at the positions of *gyrA* and *parC* that have been reported to be associated with ciprofloxacin resistance (49, 50).

### Development of cgMLST scheme.

To develop an N. meningitidis cgMLST scheme, we obtained 319 complete genomic sequences from the Assembly database of NCBI to build an allele database, using the tools developed previously (51). Genes that were present in ≥95% of the 319 genomes were assigned as core genes for N. meningitidis. The accession numbers of 319 genomes and the sequences of 1,241 core genes are listed in Table S6 and Table S7, respectively.

### Genetic relatedness analysis and strain tracking.

A total of 9,429 N. meningitidis genomic sequences were obtained from the NCBI database. For tracking the source of cc4821 and NmW:cc11 isolates from Taiwanese patients, 188 genomic sequences of the cc4821 isolates, including 1 from the United States, that were previously used in the study of Lucidarme et al. (34), an additional 17 cc4821 isolates from the United States, and 141 genomic sequences of cc11 isolates that were used in the study of Zhu et al. (35) were downloaded from the PubMLST database (https://pubmlst.org/). The genomic sequences were assembled using the SPAdes assembler (48) and converted to cgMLST profiles using the cgMLST profiling tool, provided on the website http://rdvd.cdc.gov.tw/cgMLST/. Genetic relationships among strains were established by clustering the cgMLST profiles and visualizing the genetic trees using the single-linkage algorithm or the neighbor-joining algorithm with the tools provided in the BioNumerics version 7.6 package (Applied Maths, Sint-Martens-Latem, Belgium). Strain tracking was executed by comparing a query cgMLST profile with 9,594 cgMLST profiles, using the tool installed on the website http://rdvd.cdc.gov.tw/cgMLST/.

### Data availability.

WGS reads for the 165 N. meningitidis isolates were deposited in the SRA database of NCBI, under BioProject PRJNA808787 and the accession numbers SRR18091102 to SRR18091266. The assembled contigs were also deposited in the NCBI database and the PubMLST database. The accession number for each isolate is provided in Table S5 in the supplemental material.
