# Supplementary material for: Demographic Features of Invasive Meningococcal Disease in Taiwan, 1993 to 2020, and Genetic Characteristics of Neisseria meningitidis Isolates, 2003 to 2020
Source: Microbiol Spectr. 2022 Jul 11;10(4):e00882-22. doi: 10.1128/spectrum.00882-22 (PMC9430714; doi:10.1128/spectrum.00882-22)
Supplement: Supplemental file 1 — Supplemental material. Download spectrum.00882-22-s0001.pdf, PDF file, 1.3 MB [file spectrum.00882-22-s0001.pdf]

Supplemental Figures

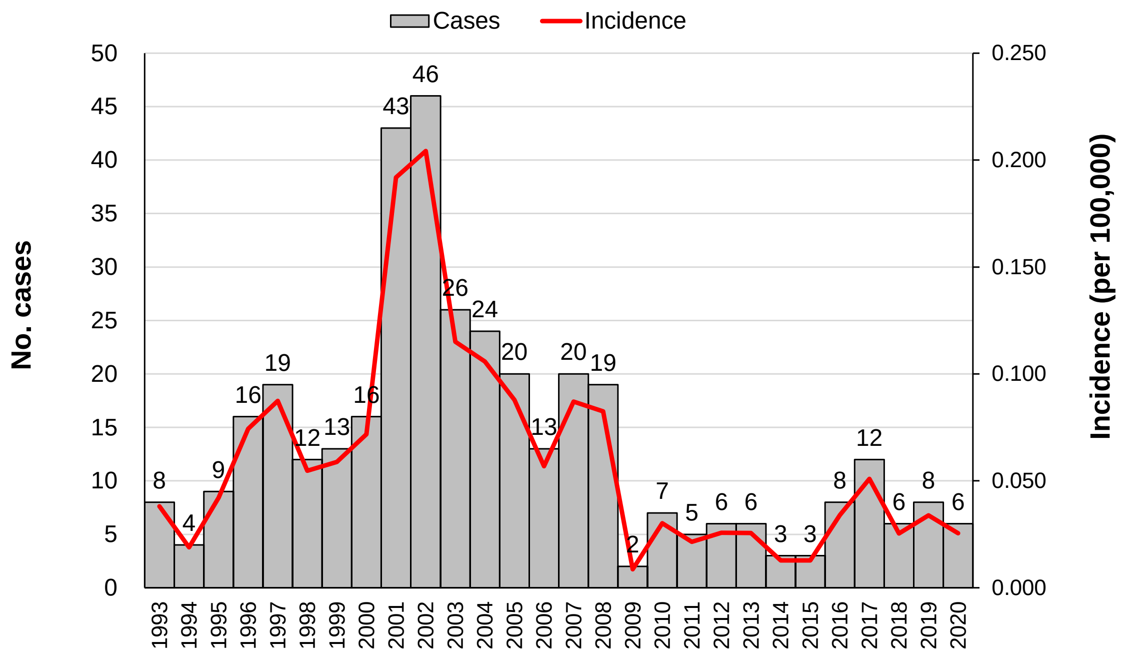

Figure S1. The annual number of cases and incidence of invasive meningococcal disease in Taiwan, 1993–2020 (n = 380).

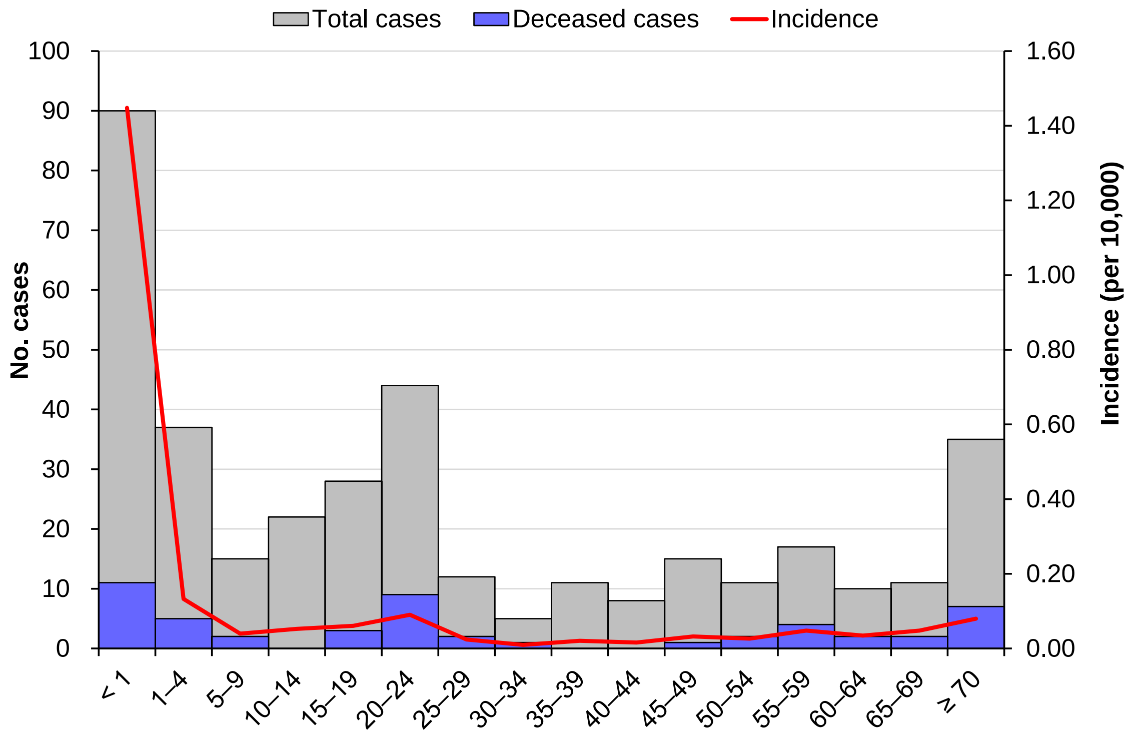

Figure S2. The number of cases and incidence of invasive meningococcal disease in Taiwan, by age group, 1993–2020 (n = 371).

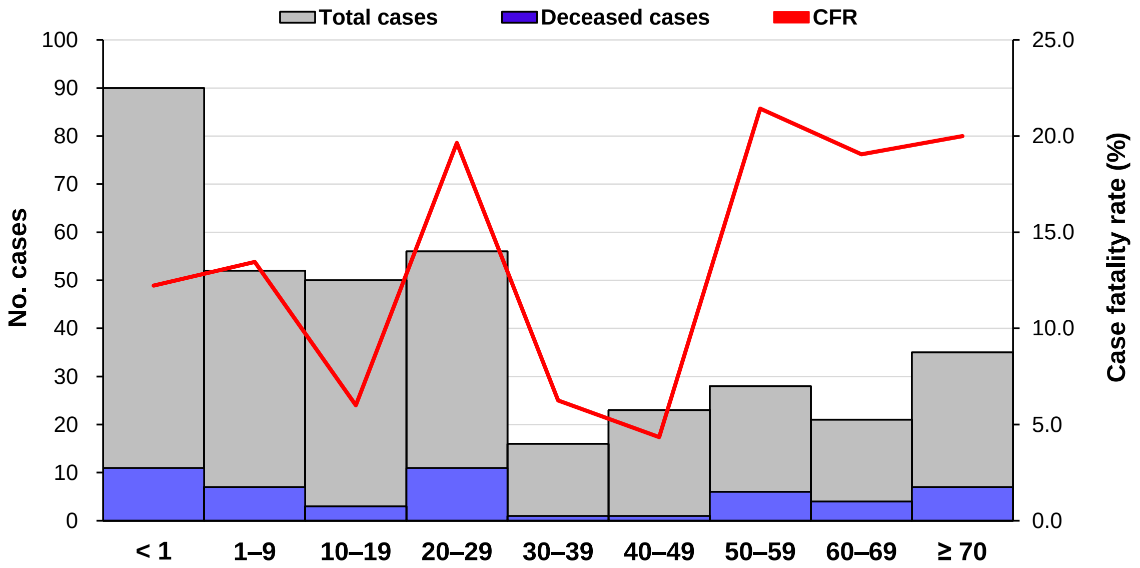

Figure S3. The number of cases and case fatality rate (CFR) of invasive meningococcal disease in Taiwan, by age group, 1993–2020 (n = 371).

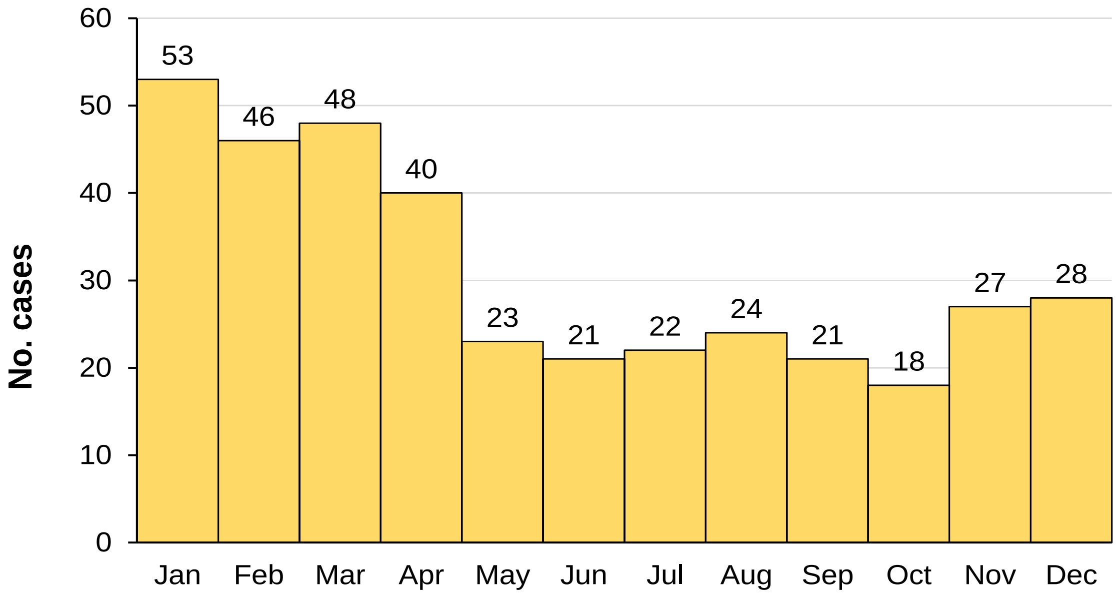

Figure S4. Cumulative cases of invasive meningococcal disease in Taiwan, by month, 1993–2020 (n = 371).

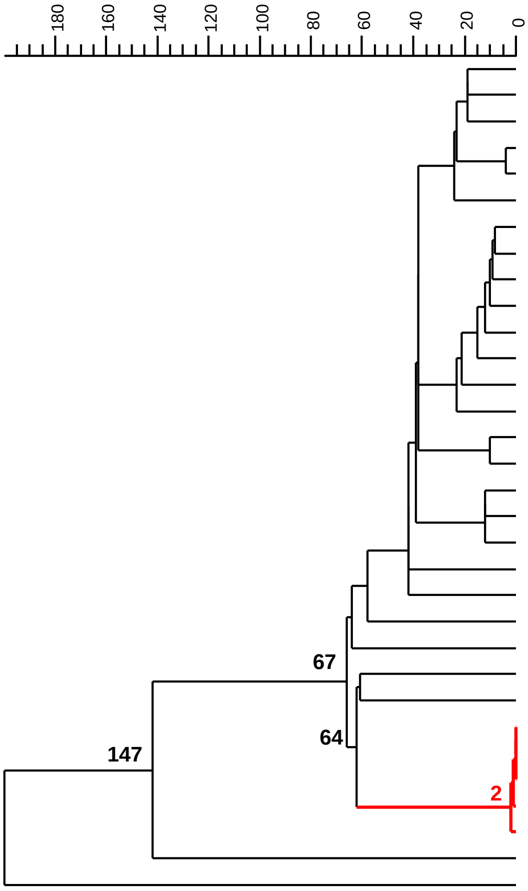

Key	Age	Sex	OnsetDate	ST	Serogroup
R18.2566	68	F	2006-11-30	3465	B
R17.0723	2	M	2010-11-09	3465	B
R18.2611	19	F	2010-06-24	3465	B
R17.0740	59	F	2016-09-17	3465	B
R20.0336	18	M	2020-01-12	3465	B
R17.0732	0	F	2013-11-16	3465	B
R18.2535	1	F	2003-11-26	3465	B
R18.2567	31	F	2006-12-22	3465	B
R18.2552	0	M	2005-03-24	3465	B
R18.2532	66	M	2003-08-21	3465	B
N93-02003	1	M	2004-02-06	3465	B
R18.2547	48	F	2005-02-27	3465	B
C05.0676	0	M	2005-04-12	5794	B
R17.0738	59	F	2016-02-21	5794	B
R17.0737	45	M	2015-10-15	5794	B
R18.2625	74	F	2018-03-25	5794	B
C06.1275	6	M	2006-06-26	3465	B
R18.2590	24	F	2007-12-19	3465	B
R18.2592	1	F	2008-01-24	3465	B
R18.2556	20	M	2005-07-01	3465	B
R17.0735	49	F	2014-01-14	3465	B
R18.2555	21	M	2005-05-02	5794	B
R17.0749	0	M	2017-03-29	3465	B
R18.2587	0	F	2007-12-03	3465	B
R18.2569	62	F	2006-12-29	3465	B
**R18.2596**	**13**	**F**	**2008-02-07**	**3465**	**B**
**R18.2599**	**14**	**M**	**2008-06-01**	**3465**	**B**
**R18.2594**	**13**	**F**	**2008-03-04**	**3465**	**B**
**R18.2604**	**1**	**F**	**2008-08-17**	**3465**	B
**R18.2597**	**13**	**M**	**2008-03-18**	**3465**	**B**
R18.2606	67	F	2008-09-30	14965	B
R20.0333	87	M	2019-08-29	11556	B

Figure S5. A cgMLST tree for 32 cc32 *N. meningitidis* isolates recovered in Taiwan, constructed using the single linkage algorithm. The isolates marked in red were from the ill students in an outbreak that occurred in a junior high school in 2008 and the one marked in blue was from a patient who was not included in the outbreak at the time.

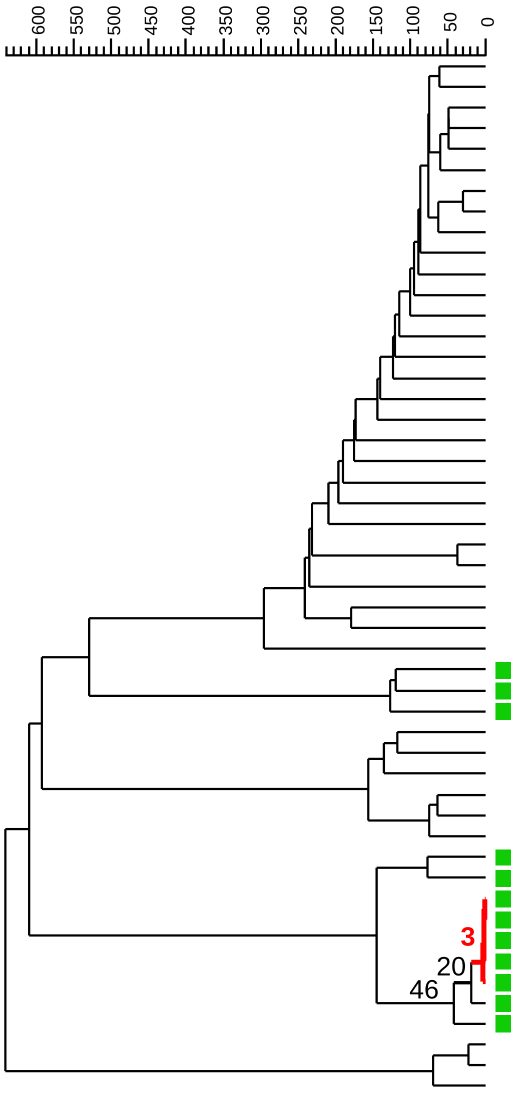

Key	Age	Sex	OnsetDate	ST	Serogroup
R18.2607	0	F	2008-11-29	3200	B
R18.2519	23	M	2003-01-20	3200	B
R18.2529	3	M	2003-06-02	3200	B
R18.2608	0	M	2009-04-06	3200	B
R18.2518	0	M	2003-01-21	3200	B
R18.2545	26	F	2005-02-13	3200	B
R17.0743	0	F	2016-10-09	3200	B
R18.2605	0	F	2008-08-26	3200	B
R18.2624	1	F	2018-03-11	3200	B
R17.0720	74	M	2010-04-15	3200	B
R17.0719	36	F	2010-01-09	3200	B
R18.2549	57	F	2005-03-22	3469	B
R17.0728	1	M	2012-11-10	14959	B
N93-03001	71	F	2004-03-08	3200	Nongroupable
R18.2610	11	F	2009-09-06	14966	B
R18.2586	2	M	2007-09-16	5798	B
R18.2573	0	M	2007-02-23	3200	B
S04.0248	29	M	2004-01-16	3200	B
NM31203	16	F	2003-03-17	3200	B
R18.2571	76	M	2007-01-22	4836	B
R18.2603	58	F	2008-08-09	15080	B
R18.2541	0	M	2004-04-20	11455	B
R18.2579	0	M	2007-02-22	3469	B
N93-04001	19	M	2004-03-29	3200	B
R18.2620	50	M	2013-12-08	3200	B
R17.0742	46	F	2016-09-26	14962	B
R18.2612	81	M	2011-01-05	3200	B
R18.2626	61	M	2018-04-08	3200	B
R18.2531	5	M	2003-08-03	3470	B
R17.0744	22	M	2016-11-17	5664	B
R17.2521	0	M	2017-05-03	5664	B
R17.0748	57	F	2017-03-24	5664	B
R18.2548	7	F	2005-03-28	5796	B
R18.2577	86	F	2007-03-18	14963	C
R17.0734	0	F	2014-01-06	14963	B
R18.2593	3	F	2008-02-20	14963	C
R17.0733	3	M	2013-11-08	14963	C
R17.0746	66	F	2017-01-27	15098	C
R17.0726	72	M	2012-03-20	4821	C
R18.2613	67	M	2011-06-15	4821	C
**R17.2522**	**20**	**M**	**2017-07-22**	**6928**	**B**
**R17.2524**	**23**	**F**	**2017-07-18**	**6928**	**B**
**R17.2523**	**22**	**M**	**2017-07-23**	**6928**	**B**
**R17.2525**	**23**	**M**	**2017-07-15**	**6928**	**B**
**R18.2622**	**45**	**F**	**2018-01-10**	**6928**	**B**
R17.0745	39	M	2016-11-22	6928	B
R20.0340	24	M	2020-05-06	6928	B
R18.2563	54	F	2006-07-13	14961	C
R20.0338	11	M	2020-02-01	14961	C
R17.0739	66	F	2016-03-12	14961	C

Figure S6. A cgMLST tree for 50 cc4821*N. meningitidis* isolates recovered in Taiwan, constructed using the single linkage algorithm. The isolates marked in red were from the patients in an outbreak that occurred in a military base in 2017 and the isolates marked in blue were from patients who were not included in the outbreak at the time. The isolates marked in green squares carry a T911 mutation in *gyrA*.

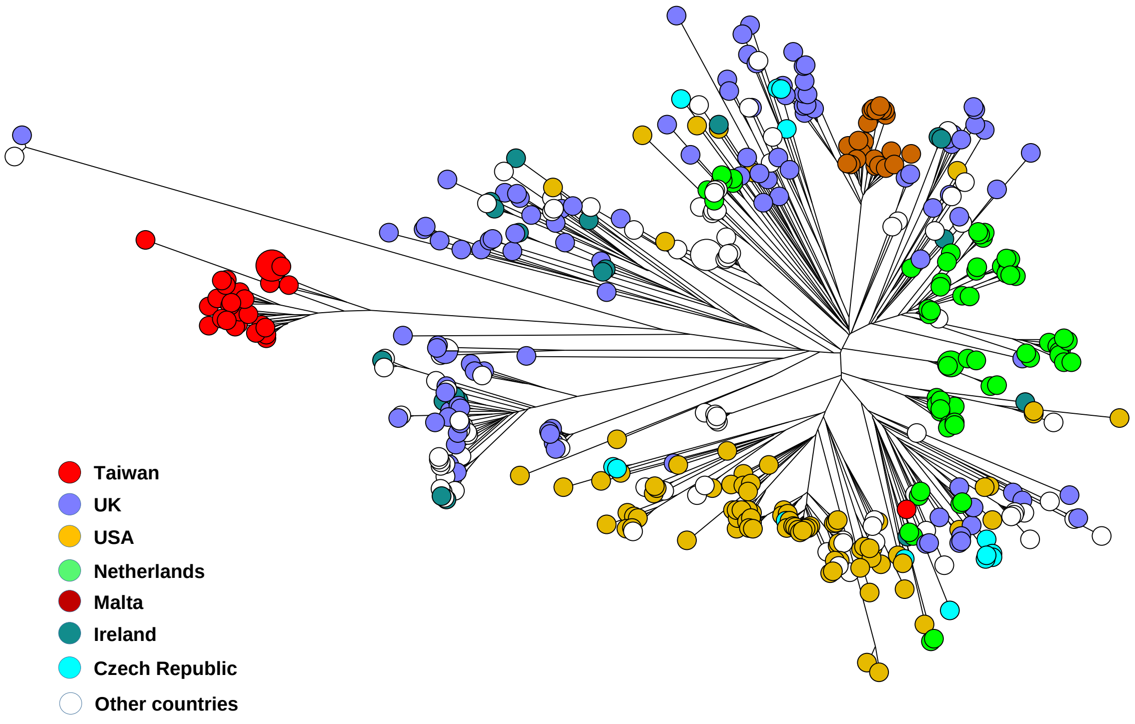

Figure S7. A cgMLST tree for the cc32 isolates from Taiwan and the NCBI database, constructed using the neighbor-joining algorithm.

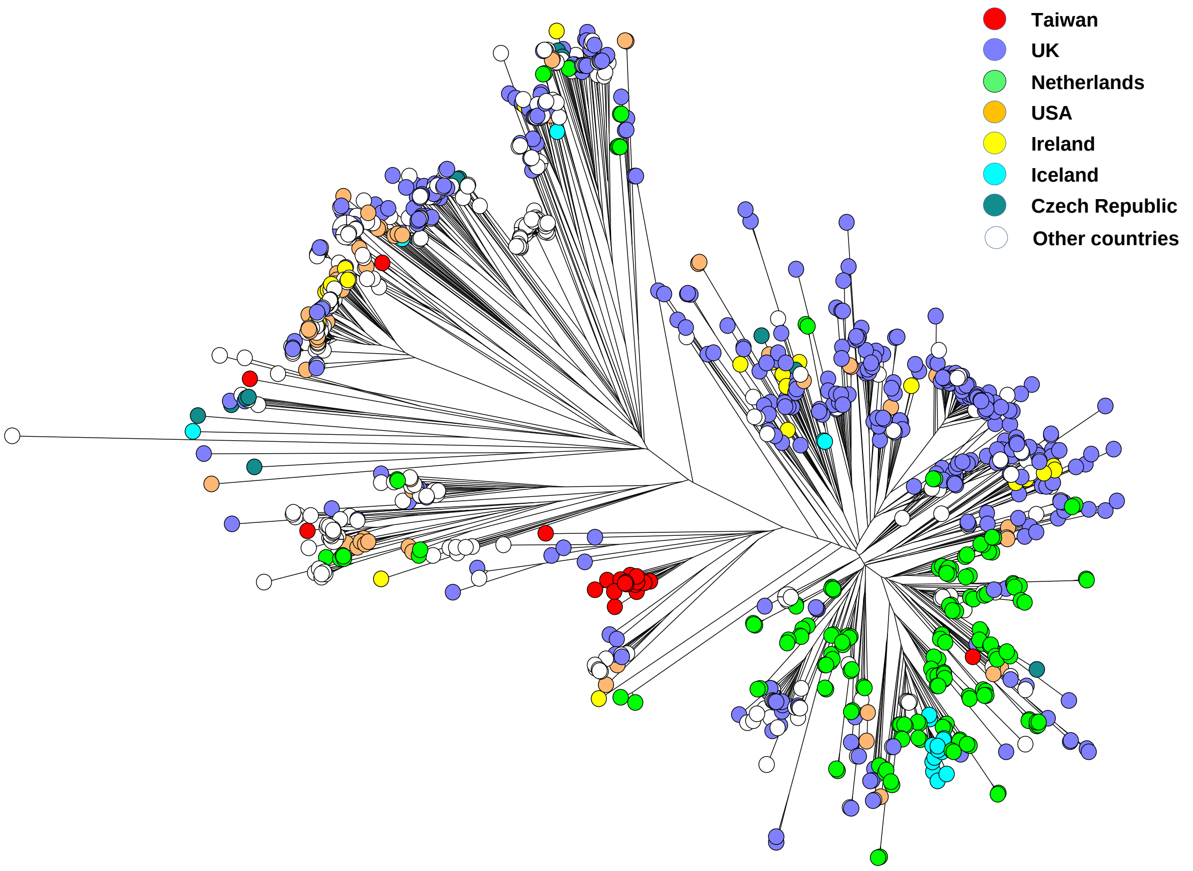

Figure S8. A cgMLST tree for the cc41/44 isolates from Taiwan and the NCBI database, constructed using the neighbor-joining algorithm.

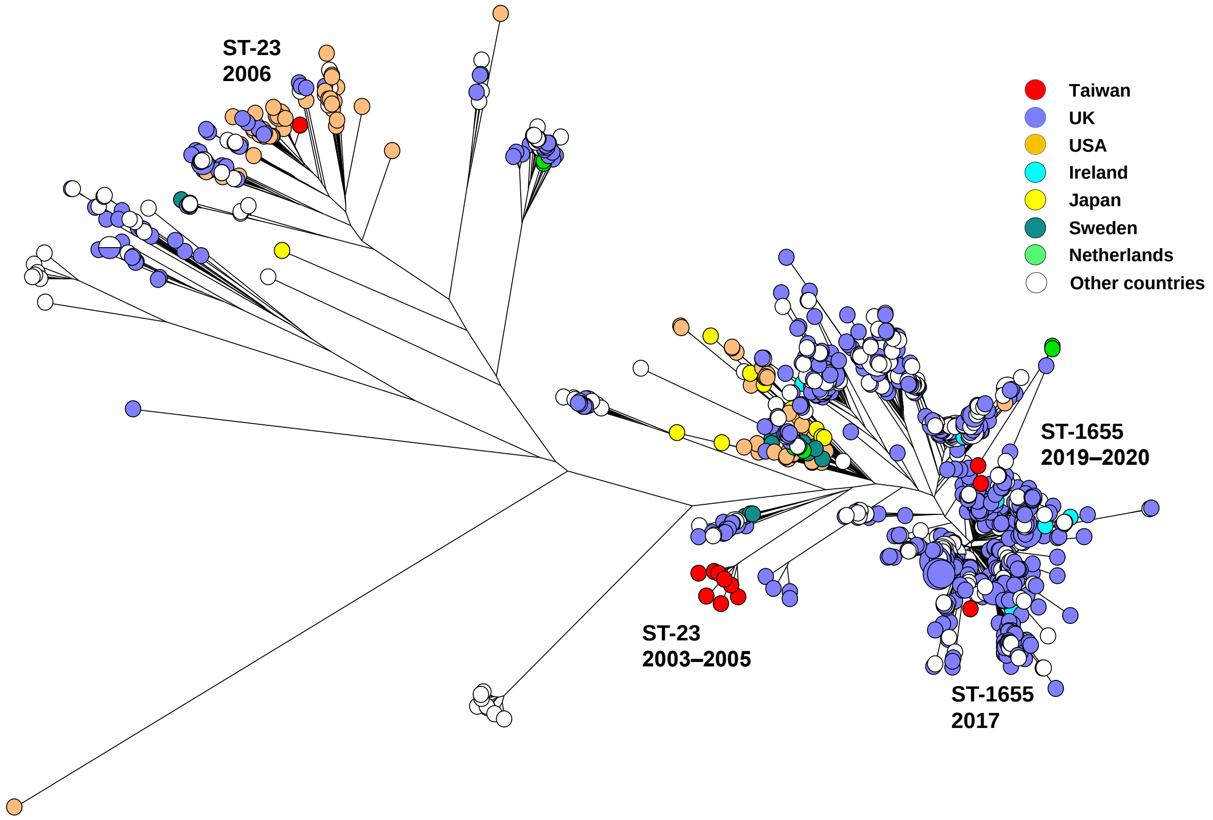

Figure S9. A cgMLST tree for the MenY:cc23 isolates from Taiwan and the NCBI database, constructed using the neighbor-joining algorithm. The year of emergence of the isolates from Taiwan is denoted.

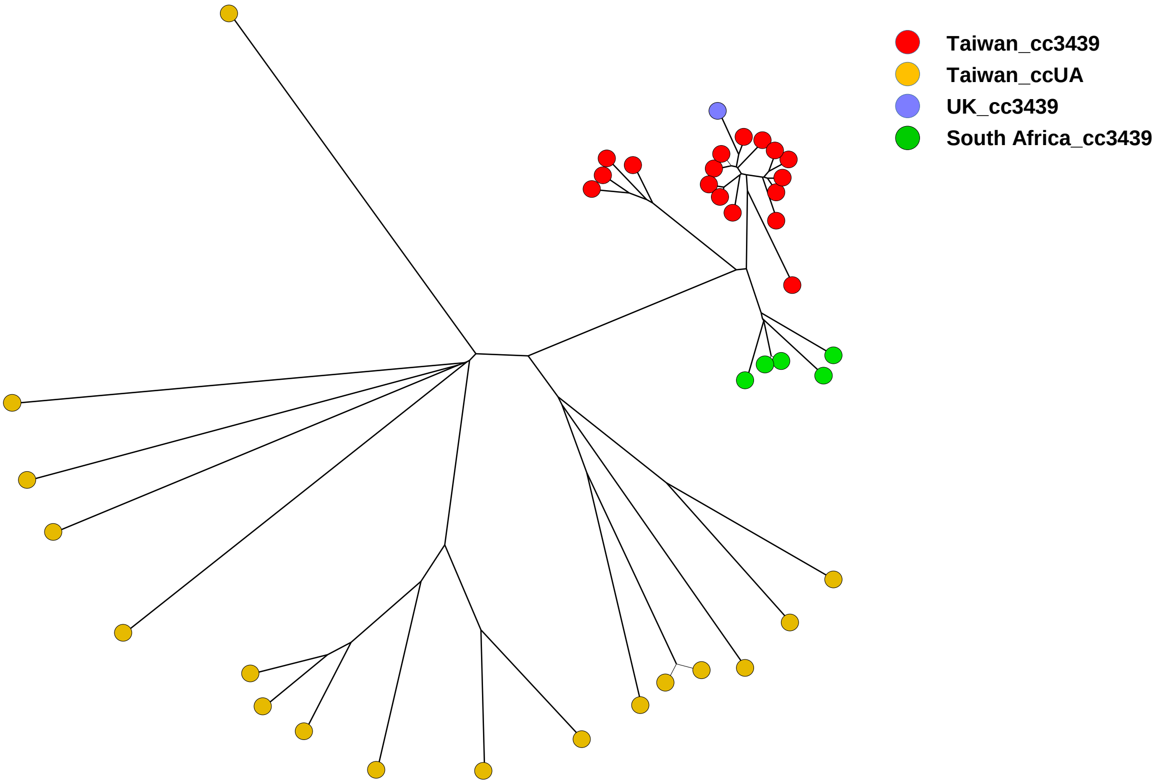

Figure S10. A cgMLST tree for the cc3439 and ccUA isolates from Taiwan and the PubMLST database (https://pubmlst.org/), constructed using the neighbor-joining algorithm.

Discussion on the TCDC and PubMLST cgMLST schemes

1. How the TCDC cgMLST scheme was developed?

The TCDC cgMLST scheme was developed using a computational tool, which includes the Prodigal [1] and the Roary [2] tools to predict open reading frames from a genome set (319 genomes) and to perform clustering analysis of ORFs; each ORF cluster is subsequently defined as a gene or a locus. The frequency of each gene (locus) over the 319 genomes was calculated and the most frequent peptide sequence (mode) of each locus was selected as the reference sequence of the locus. Among the loci, 1,241 which were identified to be present in ≥ 95% of the 319 genomes were assigned to be the core genes of *Neisseria meningitidis* (for the distribution of frequency of genes, please see Figure A).

The 319 genomes were obtained from the National Center for Biotechnology Information (NCBI) database (https://www.ncbi.nlm.nih.gov/). The NCBI accession numbers for the 319 genomes and the peptide and nucleotide sequences of 1,241 core genes can be found in Supplementary file 1, Table S6, and Table S7, or the website: http://rdvd.cdc.gov.tw/cgMLST/. (Note: This website is established in Taiwan CDC and could be temporarily shut down to defend against massive cyber-attacks).

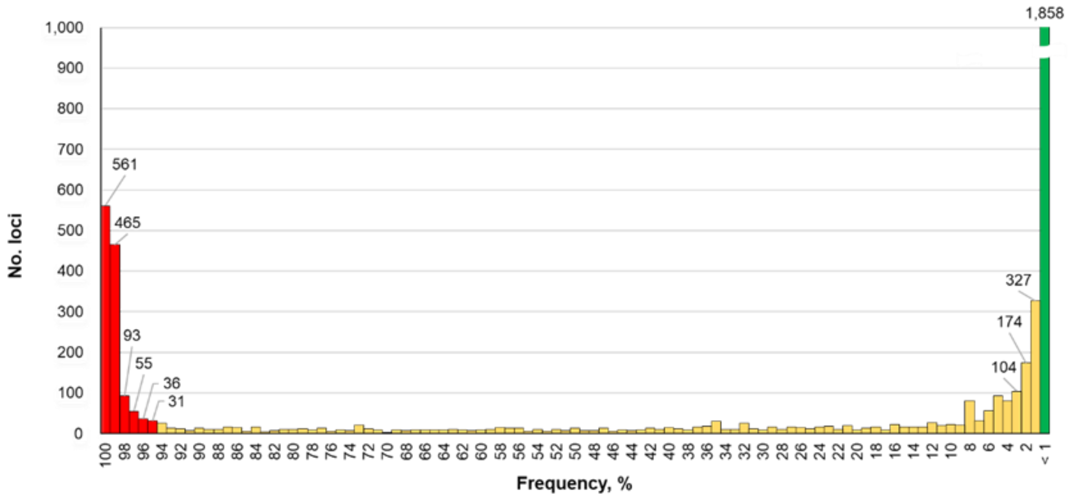

Figure A. Frequency of loci (genes) over 319 *N. meningitidis* genomes.

2. The diversity of the reference panel (n=319) of isolates used to develop the scheme and how it compares to the panel used by Bratcher et al [3] for the existing scheme.

Phylogenetic analysis indicates that the genomes used for the PubMLST scheme and the Taiwan CDC (TCDC) scheme are distributed in all major lineages (Figure B). However, the PubMLST scheme has a higher proportion of genomes in lineage A and the TCDC scheme has more number of closely related strains in lineage B.

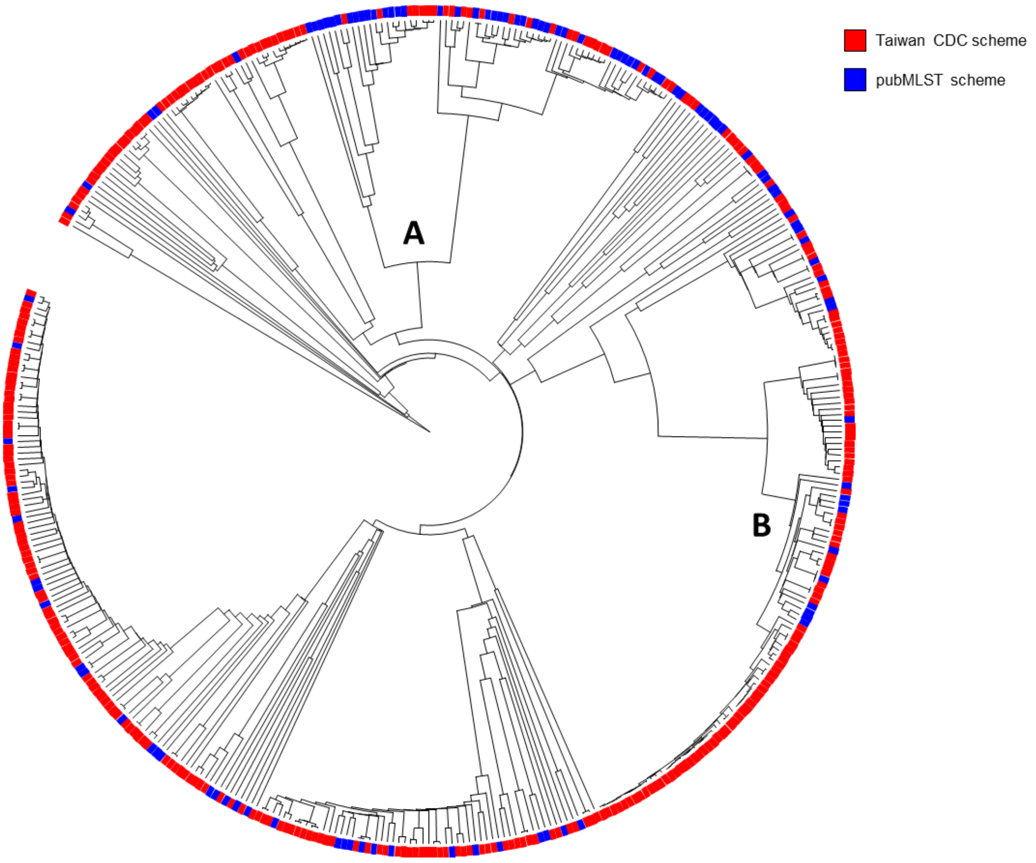

Figure B. Phylogenetic tree for the *N. meningitidis* strains used in the development of the PubMLST scheme and the TCDC scheme. The tree was constructed using the Mash (K-mer) algorithm [4].

3. The loci that were included and not included in the PubMLST scheme and their % representation among the isolate/genome panel used to develop the scheme.

Among the 1,605 PubMLST loci, 1,537 are included among the TCDC loci identified from the 319 genomes, which are corresponding to 1,526 of the TCDC loci (including non-core genes), and 68 loci are not unidentified. Table A shows that 1,208 loci could be the common core genes in both schemes, while 318 corresponding loci were not designated as core genes in the TCDC scheme because the loci have a frequency of <95% over the 319 genomes. However, it is difficult to precisely compare the genes between the two schemes because a gene in one scheme may correspond to multiple genes in the other. As indicated in Table B, for some loci in the TCDC scheme, each could correspond to 2-3 loci in the PubMLST scheme.

Table A. The frequency and the number of the TCDC loci matched with the PubMLST loci.

Frequency over 319 genomes	No.TCDC loci matched with PubMLST loci
95-100	1208
90-94	60
80-89	74
50-79	118
20-49	55
<20	11
Total	1526

Table B. Locus in the TCDC scheme has multiple corresponding loci in the PubMLST scheme.

Taiwan CDC locus_id	PubMLST locus id	Frequency
spuD_2	NEIS1516, NEIS1689	100
spuD_1	NEIS1516, NEIS1689, NEIS0567	100
dsbA_2	NEIS1885, NEIS0273	100
group_4984	NEIS0952, NEIS1663	99.69
ackA_1	NEIS1727, NEIS1447	99.69
group_172	NEIS1662, NEIS0951	99.37
group_582	NEIS0795, NEIS0524	97.49
group_5078	NEIS1665, NEIS0954	97.18
group_1010	NEIS0608, NEIS0595	96.24
mafA1_1	NEIS1789, NEIS2083	89.97
mdaB_1	NEIS0361, NEIS0957	87.77
tufA_2	NEIS0128, NEIS0116	84.95
group_5110	NEIS1664, NEIS0953	75.86
group_308	NEIS1795, NEIS1796	52.98
rfbC	NEIS0065, NEIS0045	47.65
group_22	NEIS0950, NEIS1661	44.51
group_556	NEIS0779, NEIS0777	34.8
group_1397	NEIS1800, NEIS0601, NEIS0599	31.66
group_2183	NEIS1800, NEIS0601	0.94

4. One gene could be split into multiple loci in the PubMLST scheme.

   For example, NEIS1800, NEIS0601, and NEIS0599 are corresponding to locus “group_1397” in the TCDC scheme. Clustering analysis of the peptide sequences of the alleles of NEIS1800, NEIS0601, and NEIS0599 indicated that the alleles of each of the 3 loci were mixed but not distinctly separate (Figure C). Thus, the 3 loci could belong to a common gene.

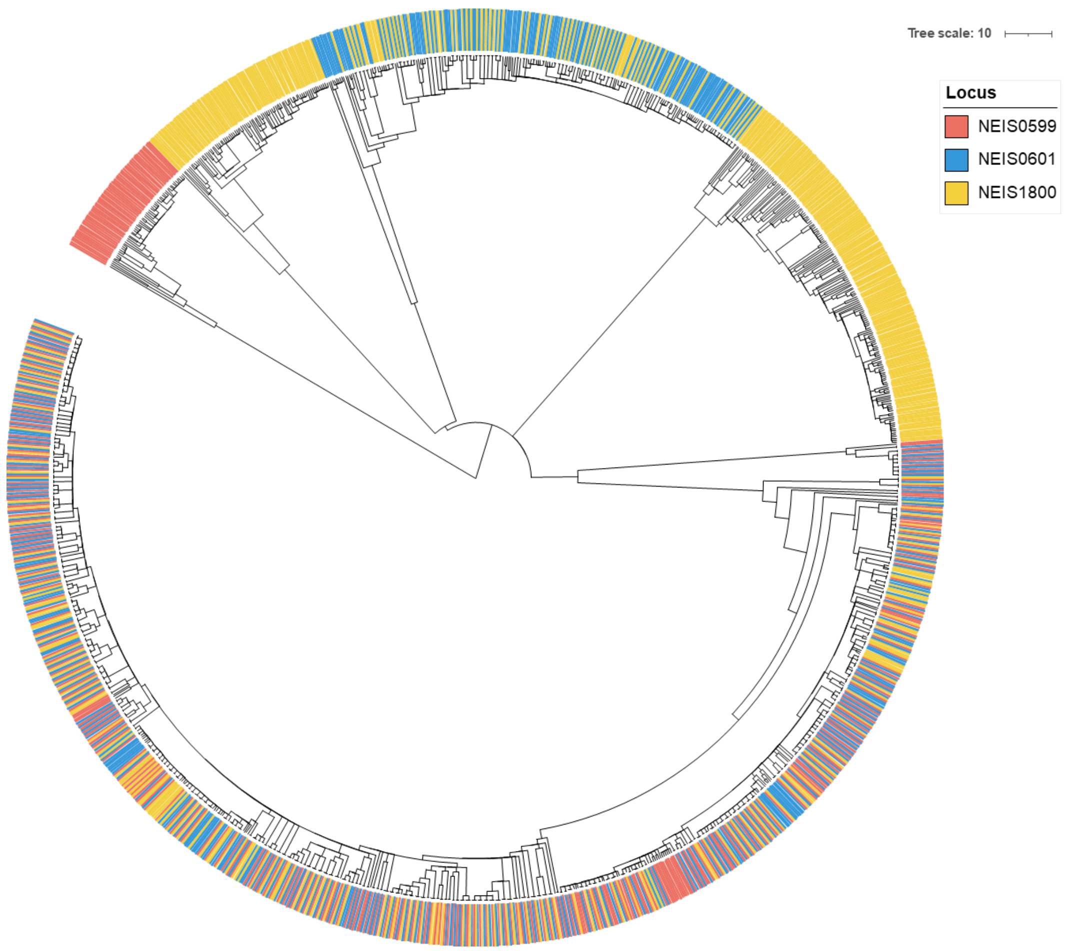

Figure C. Clustering analysis of the peptide sequences of alleles of the NEIS1800, NEIS0601, and NEIS0599 loci in the PubMLST database.

5. How the TCDC scheme differs from the existing PubMLST scheme

How to define a gene could be critical, whereas different sets of genomes used in developing a cgMLST scheme would affect the frequency of genes over the genome set in assigning core genes. The TCDC scheme was developed using the computational programs including the tool Prodigal [1] to predict ORFs from genomic sequences and the Roary [2] to perform clustering of the total ORFs from the genome set. An ORF cluster is defined as a gene (locus). The cutoff value for defining a gene in the ORF clustering was set at ≥95% peptide sequence identity and ≥75% length coverage. Different genome sets could affect the frequency of genes over the genomes in designating core genes. For the TCDC scheme, core genes were assigned for those existing in ≥95% of the genome set. As mentioned above, for some loci, a TCDC locus could be responding to multiple (2-3) PubMLST loci, indicating that a gene defined in the

TCDC scheme could be possibly split into 2-3 loci in the PubMLST scheme.

6. Why the tools used in the development of the TCDC scheme failed to identify 68 genes previously identified by Bratcher et al? [3]

   The reasons could be that the ORFs of the 68 genes were not predicted from the 319 genomes by the rules set by the Prodigal or the ORFs had been clustered into other gene families (having ≥95% identity with other genes). For example, among the 68 genes, locus NEIS0154 is the 50S ribosomal protein L36, having 37 aa, but in the TCDC scheme, the gene of 50S ribosomal protein L36 has 41 aa. Both sequences share only 53% identity.

7. What is the performance of both schemes in strain tracking and comparing genetic relatedness of isolates?

   Both schemes were compared by generating cgMLST profiles of 334 genomes retrieved from the PubMLST database. The profiles generated using the PubMLST scheme had more void loci (genes not detected) than those generated by the TCDC scheme (with an average of 76.9 vs. 14.7 void loci/genome) (Figure D). This can be explained partly as the PubMLST scheme has more loci than the TCDC scheme. However, 95.6% (1,186/1,241) of the TCDC loci are existing in ≥95% of the 334 genomes, while only 78.9% (1,266/1,605) of the PubMLST loci are existing in ≥95% of the genomes (Table C), indicating that some loci of the PubMLST scheme should not be true core genes or not be existing in the majority of *N. meningitidis* strains.

   Table C also indicates that 31 of the 68 loci, which are not found in the TCDC scheme, are existing in ≥95% of the genomes. Thus, some loci unidentified among the TCDC loci could be existing but were not identified by the tools used for developing the TCDC scheme, or some of the 68 loci have been incorporated into the core genes in the TCDC scheme.

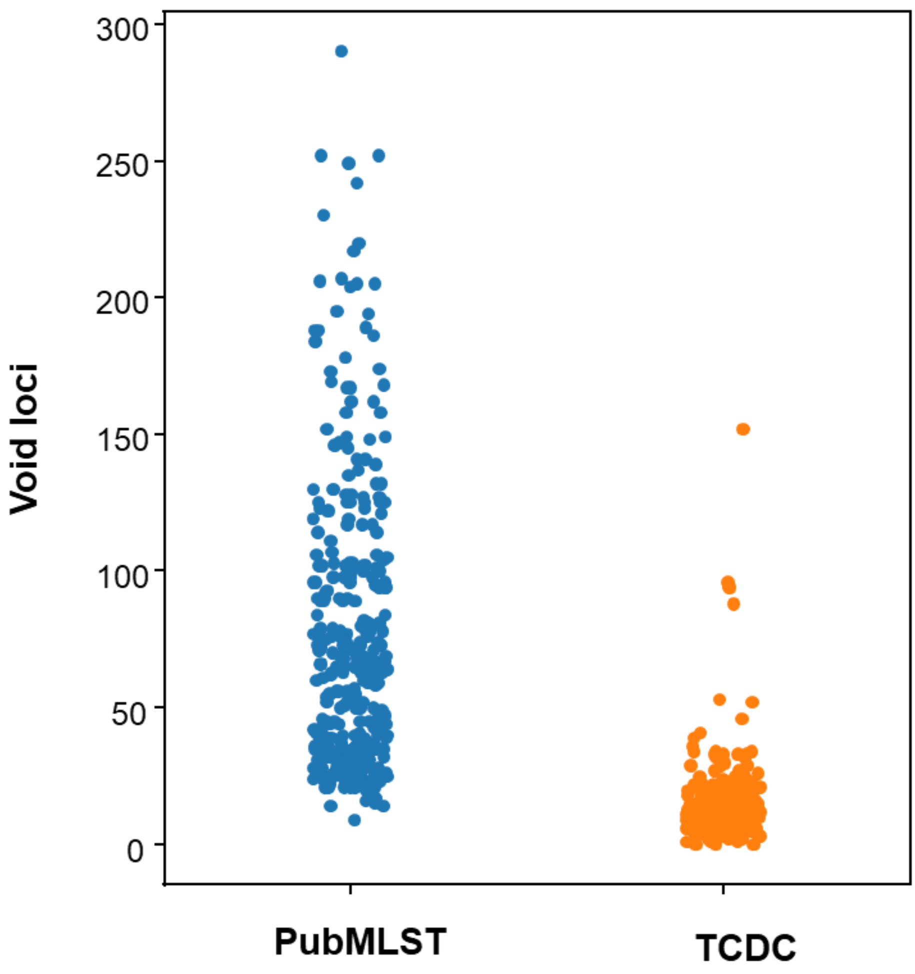

Figure D. Distribution of void loci in cgMLST profiles generated from 334 genomes using the PubMLST and TCDC schemes (the 334 genomes were obtained from the PubMLST database).

Table C. Frequency and number of loci identified in 334 genomes using the PubMLST and TCDC cgMLST schemes.

Frequency	PubMLST	TCDC	Loci not found in TCDC
100	229	586	10
95.0-99.9	1,037	600	21
80.0-94.9	259	37	18
50.0-79.9	43	15	5
20.0-49.9	31	3	10
0-19.9	6	0	4
Total	1,605	1,241	68

8. Should the allele database for the TCDC scheme need to be updated over time?

   The TCDC scheme does not need an allele database, it does not need to update new alleles over time. Of the scheme, the most common peptide (mode) of each locus (gene) was selected as the reference (representative) of the locus. A set of peptide references for the core genes are used for cgMLST profiling. In cgMLST profiling, OFRs from a genome are compared with the references, then the corresponding nucleotide sequences of matches (≥95% amino acid sequence identity and ≥75% coverage with the references) are converted into SHA256 codes. Therefore, a cgMLST profile of a genome is an array of SHA256 codes in order. A cgMLST profile of a genome is always identical whenever it is generated. Thus, no allele database is needed for cgMLST profiling and no new alleles need to be updated over time. In the TCDC method, only a set of reference sequences is needed for cgMLST profiling. The probability of collision using SHA256 codes is 10^{-77} (2^{-256}).

References

[1] Hyatt D, Chen G-L, Locascio PF, Land ML, Larimer FW, Hauser LJ. Prodigal: prokaryotic gene recognition and translation initiation site identification. BMC Bioinformatics 2010;11:119-. doi: 10.1186/1471-2105-11-119.

[2] Page AJ, Cummins CA, Hunt M, Wong VK, Reuter S, Holden MT, et al. Roary: rapid large-scale prokaryote pan genome analysis. Bioinformatics 2015;31:3691-3. doi: 10.1093/bioinformatics/btv421.

[3] Bratcher HB, Corton C, Jolley KA, Parkhill J, Maiden MC. A gene-by-gene population genomics platform: de novo assembly, annotation and genealogical analysis of 108 representative *Neisseria meningitidis* genomes. BMC Genomics 2014;15:1138. doi: 10.1186/1471-2164-15-1138.

[4] Ondov BD, Treangen TJ, Melsted P, Mallonee AB, Bergman NH, Koren S, et al. Mash: fast genome and metagenome distance estimation using MinHash. Genome Biol 2016;17:132. doi: 10.1186/s13059-016-0997-x.
